# Immunotherapy Responsive Autoimmune Subacute Encephalitis: A Report of Two Cases

**DOI:** 10.1155/2010/837371

**Published:** 2010-03-18

**Authors:** Manoj Mittal, Nancy Hammond, Sharon G. Lynch

**Affiliations:** Kansas University Medical Center, 5508 W 70th Street, Prairie Village, Kansas City, KS 66208, USA

## Abstract

*Objective.* To describe the clinical characteristics and radiological findings in two patients with subacute encephalitis associated with elevated serum voltage-gated potassium channel antibody (VGKCAb) and antithyroperoxidase (TPO) antibody. *Case Reports. *Case 1: 63-year-old woman was admitted for altered mental status and possible seizure activity. MRI brain showed hyperintensity in the bilateral hippocampal areas. She was positive for VGKCAb and anti-TPO antibodies. She was treated with steroids, IVIG, plasma exchange and azathioprine. After 8 months, she had marked improvement in her memory and seizures. Case 2: 61-year-old woman was admitted for video EEG monitoring of unclassified seizure and cognitive function decline. MRI of the brain showed mild hyperintensity in bilateral hippocampal areas and significant atrophy in the frontotemporal lesion. Anti-TPO antibody and VGKCAb were positive. She was treated with steroids, plasma exchange and azathioprine. After 9 months, she had marked improvement in her memory and seizures. *Conclusion.* Autoimmune subacute encephalitis appears to be an underdiagnosed entity. It is important to screen patients with subacute encephalitis for anti-TPO antibody and VGKCAb, particularly in the presence of seizures. Immunosuppressive therapy appears to be effective in treating this entity.

## 1. Introduction

Subacute encephalopathies are neurological and systemic diseases causing impairment of consciousness insidiously over weeks or months. Hashimoto's encephalitis (HE) is a neurological disorder associated with elevated levels of antithyroperoxidase (TPO) antibody, antithyroglobulin (anti-TG), antiNH2-terminal of a-enolase (NAE) antibody or anti thyrotropin antibodies. HE responds to treatment with steroids or plasma exchange and has a good prognosis [1, 2]. Voltage-gated potassium channel antibody (VGKCAb)-associated encephalitis is a paraneoplastic or nonparaneoplastic disorder characterized by amnesia, delirium, and seizures with good response to intravenous immune globulin (IVIG), plasma exchange, and steroids [3–7]. We present 2 patients with subacute encephalitis who were positive for both antibodies.

## 2. Case Report

### 2.1. Case 1

**Figure 1:**
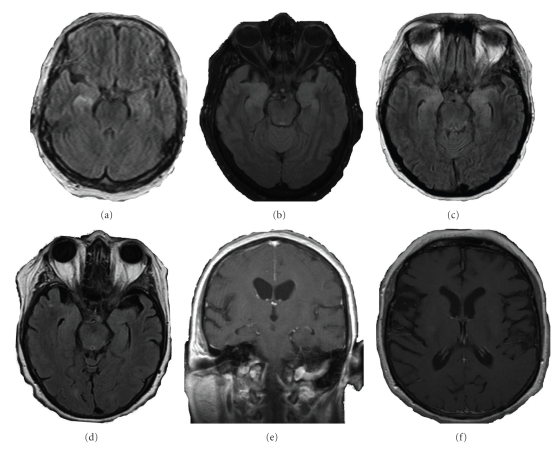
MRI findings in two cases of immunotherapy responsive autoimmune subacute encephalitis. Brain MRI of Patient 1 ((a), (b)) with immunotherapy responsive voltage-gated potassium channel antibody and antithyroperoxidase antibody-associated subacute encephalitis illustrates increased signal in the bilateral medial temporal lobes on T2 fluid attenuated inversion recovery (T2 FLAIR) sequences ((a) initial image). Follow up image after 8 months (b) was unchanged. Brain MRI of case 2 illustrates increased signal in the bilateral medial temporal lobes on T2 FLAIR ((c) initial image) and after 13 months (d) shows its resolution; T1 with contrast sequence ((e) initial image) shows bilateral temporal atrophy and after 13 months (f) showing persistent bilateral frontotemporal atrophy.

63-year-old, right-handed woman admitted for altered mental status and possible seizure activity. Seizure activity involved twitching of right face, a flexion motion of the elbow and stiffening of the arm, lasting around 20 seconds. She also had one episode of generalized tonic clonic seizure. She had a hypomanic state, visual hallucinations, short-term memory problems, loss of social inhibition and history of multiple falls. Past medical history was significant for untreated hypothyroidism. On examination, the patient was oriented to person only. She had receptive aphasia and perseveration. Gait was ataxic. The video EEG showed marked bifrontal and bitemporal slowing with electrographic seizures originating from the left temporal lobe. Treatment with levetiracetam resulted in initial improvement. Worsening in 3 days required the addition of valproic acid. MRI brain showed hyperintensity on T2 flair images in the bilateral hippocampal areas, right more than left (Figure 1). CSF showed 10 white blood cells (WBCs), glucose 49, and protein 44. Her CSF protein 14-3-3 and herpes simplex virus polymerase chain reaction (HSV PCR) were negative. Serum paraneoplastic panel was positive for VGKCAb of 6.21 (reference range (RR): ≤0.02 nmol/L). Her serum thyroid stimulating hormone (TSH) was 6.35 (RR: = 0.35–5.00 mcu/ml) and antiTPO antibodies were 142 (RR: = 0–35 iu/ml). Serum mean sodium was 135 ranging from 128 to 140 mmol/L. Cancer work up was negative (CT chest, abdomen and pelvis, mammogram, colonoscopy and whole body PET scan). 

She was treated with synthroid 50 mcg daily and IV methylprednisone 1000 mg daily for 5 days followed by IVIG and plasma exchange with no improvement in mental status. She was discharged on prednisone and azathioprine. At 8 months follow up, she had marked improvement in her memory, seizures, and walking. VGKCAb was reduced from 6.21 to 0.86 nmol/L and antiTPO antibody had gone down from 142 to 32.4 iu/ml. Repeat MRI 8 months after the initial MRI was unchanged.

### 2.2. Case 2

61-year-old right-handed woman was admitted for video EEG monitoring of unclassified seizure. She had 6 months history of frequent, brief episodes of fearful waking, associated with stiffness and moving her right arm towards her body, grabbing her elbow and asking repeatedly “Help me. Help me. Where are they?” The patient's family reported a decline in the patient's cognitive functions over the last few months and a tendency to talk aloud to herself. She had been recently diagnosed with syndrome of inappropriate antidiuretic hormone (SIADH) which was treated with demeclocycline. On examination the patient was oriented to person only. She had word finding difficulty and mild-moderate cognitive linguistic deficits in areas of attention, memory, complex reasoning, and problem solving tasks. Gait was ataxic. Video EEG monitoring showed mild diffuse slowing but no epileptiform discharges. She was started on levetiracetam with a significant decrease in seizure frequency. MRI of the brain showed mild hyperintensity in bilateral hippocampal areas on T2 flair sequences and significant atrophy in the frontotemporal lesion (Figure 1). CSF analysis showed 50,500 red blood cells (RBCs), 87 WBCs, glucose 59, and protein 77. Her CSF protein 14-3-3 and HSV PCR were negative. AntiTPO antibody (474 iu/ml), antithyroglobulin (102 [RR = 0–40 iu/ml]) antibody, and VGKCAb (0.13 nmol/L) were elevated. Cancer work up was negative. She was started on valproic acid and high-dose IV methylprednisone and, within 3 to 4 days, the patient showed significant seizure control with slight improvement in cognition and memory. Her antiTPO antibody decreased to 474 iu/ml. She was readmitted 2 months later for increased seizure frequency, myoclonic jerks, and worsening cognition. She received 5 cycles of plasma exchange with no change in cognition. The patient was discharged on prednisone and azathioprine. Follow up at 9 months showed no seizure activity and marked improvement in mental status with some deficits in short-term memory. Her SIADH resolved. Repeat MRI, 13 months after the initial MRI, showed persistent atrophy in frontotemporal region but resolution of the hyperintense lesions in the bilateral hippocampal areas (Figure 1). VGKCAb level decreased from 0.13 to 0.01 nmol/L and antithyroglobulin decreased from 102 to 53 iu/ml after 15 months on immunotherapy.

## 3. Discussion

Nonparaneoplastic limbic encephalitis (LE) has been reported in association of both antiTPO antibody and VGKCAb in 2 patients by Thieben et al. [7] and 1 patient by McKnight et al. [5] (Table 1). Similarly, both of our patients had elevated VGKCAb, and antiTPO antibody. Clinical features of both are similar to CJD including seizures, behavioral and psychiatric manifestations, movement disorders, and coma [2, 3]. Resemblance in presentation of both antibody syndromes makes it hard to identify primary antibody causing LE. VGKCAbrelated LE has been associated with levels as low as 0.03 nm/L [6]. Both of our patients had VGKCAb levels significantly higher than 0.03. Anti-NAE autoantibodies have been found to be a specific marker for HE in 44% of patients [8]. We did not check our patients for Anti-NAE autoantibodies. Hyponatremia is often seen in VGKCAbrelated LE [2, 3, 7]. Our first patient had variable sodium, with levels as low as 128. The second patient was diagnosed with SIADH requiring demeclocycline treatment. The sodium abnormalities resolved along with their encephalitis. Brain MRIs in patients with VGKCAb are reported to have hyperintensity in the hippocampus or amygdala [3]. In HE, the pattern is of atrophy and sometimes white matter hyperintensity. Our first patient's scan was more suggestive of the pattern reported in VGKCAb. Patient 2 had fronto-temporal atrophy, more suggestive of HE [9], although there was a subtle increase in intensity in the hippocampus in FLAIR images, more suggestive of those reported in VGKCAb [3, 5, 7]. VGKCAb has been associated with cancer (lung, breast, prostrate, thymoma and hematological malignancy) in some patients [6, 10]. Most of the cancers presented within 4–6 months after presentation of limbic encephalitis (range, 1–48 months) [7]. None of our patients had any evidence of cancer at 12–15 months follow up. The presence of cancer indicates poor prognosis for recovery. 

Treatment options include intravenous steroids, intravenous immunoglobulin or plasma exchange for acute presentations followed by maintenance immune therapy (oral prednisone, IVIG, or immunosuppressants) [3–7]. Treatment may have early response or may take 3–8 weeks before any improvement in cognition. Patient 2 had mild immediate improvement in cognition and memory following intravenous steroids and later following plasma exchange while patient 1 did not show any early improvement. Both of our patients had slow, progressive improvement in their cognition and memory without any relapse while on prolonged steroids and azathioprine. Clinical improvement in both of our patients was associated with a decline in the levels of VGKCAb and antiTPO antibody. MRI lesions were unchanged in patient 1 at 8-month follow up and resolved in patient 2 at 13-month follow up, indicating that it may take 9 months or more for the MRI lesions to clear up [11]. Patients with VGKCAbrelated LE may have a relapsing course [3]. Patient 2 had relapsed when treated only with a short course of steroids. VGKCAb and antiTPO antibody-related encephalitis is a treatable entity with good prognosis ranging from 60%–92% [3, 7]. Autoimmune subacute encephalitis appears to be an underdiagnosed entity [12]. In our opinion, coexistence of antiTPO antibody and VGKCAb indicates a broader autoimmune abnormality, with multiple autoantibodies still undiscovered. Patients with a diagnosis of HE or VGKCAb may be tested for a broader range of antibodies in order to clarify this relationship. Anti-TPO antibodies may not be pathogenic [13]. In this case, the presence of other autoantibodies should be sought in cases of subacute encephalitis in patients with antiTPO antibodies alone. As our knowledge of the so-called paraneoplastic antibodies expands, we may find that the autoantibodies actually responsible for the encephalitis might be quite different from those that have already been discovered. AntiTPO antibodies or VGCKAb may actually represent an epiphenomenon rather than a true causative factor. 

##  Disclosure of Any Financial Interests

Dr. Manoj Mittal and Dr. Nancy Hammond report no disclosures. Dr. Sharon G. Lynch received research support from pharmaceutical companies (Actelion, Artielle, Bayer, Biogen, Cephalon, Elan, Eli-Lily, Genentech, Genzyme, Keva, Novartis, and UCB) and National Multiple Sclerosis Society.

## Figures and Tables

**Table 1:** Comparison of reported cases of subacute encephalitis associated with voltagegated potassium channel antibody (VGKCAb) and antithyroperoxidase (TPO).

Reference no./age/sex	Seizure type	Initial S. sodium (meq/l)	Serum VGKC Ab level (nmol/L)	Other antibodies	MRI^Ψ^	EEG^Φ^	Response to immune therapy*	Outcome**	Follow up MRI***
Case 1/63/F	CPS, GTCS	128	6.21	TPO, 142	MTS (R > L)	F,T,E	Good (1, 2, 3)	M, S	R (right), N
Case 2/61/F	Frontal lobe seizure (CPS), GTCS	121	0.13	TPO, 474	MTS, A	D, B	Good (1, 3)	M	R
				Anti-TG,102					
[7]/49/F	Probable CPS	127	1.42	TPO, 45	MTS	D, T	Good (1)	M	R
[7]/65/M	CPS GTC	120	3.30	TPO, 118; GAD65, 0.09	MTS	D	Good (1)	M	R
[5]/36/F	GTCS	NA	1.038	TPO	N	NA^§^	Spontaneous resolution	Good	NB

^Ψ^MTS = Bilateral mesial temporal high signal, A = bilateral frontotemporal atrophy, N = Normal.

^Φ^D = Diffuse slowing, E = bitemporal epileptiform discharges, B = Frontal beta activity, T = bitemporal slowing, F = bifrontal slowing.

*1 = Intravenous steroids, 2 = Intravenous immunoglobulin (IVIG), 3 = Plasma exchange (PLEX), 4 = Azathioprine, 5 = Rituximab, 6 = Mycophenolate mofetil.

**M = Memory deficits, B = Behavioral changes, S = Seizures.

***H = Hippocampal atrophy, R = Resolution of high signals, NB = Normal at baseline, N = new lesion in the left medial temporal lobe.

^§^NA = Data not available.
